# Differential Expression of Serum MicroRNAs Supports CD4^+^ T Cell Differentiation into Th2/Th17 Cells in Severe Equine Asthma

**DOI:** 10.3390/genes8120383

**Published:** 2017-12-12

**Authors:** Alicja Pacholewska, Matthias F. Kraft, Vincent Gerber, Vidhya Jagannathan

**Affiliations:** 1 Department of Clinical Veterinary Medicine, Swiss Institute of Equine Medicine, Vetsuisse Faculty, University of Bern and Agroscope, Länggassstrasse 124, 3012 Bern, Switzerland; alicja@rth.dk (A.P.); matthias.kraft@vetsuisse.unibe.ch (M.F.K.); vinzenz.gerber@vetsuisse.unibe.ch (V.G.); 2 Department of Clinical Research and Veterinary Public Health, Institute of Genetics, Vetsuisse Faculty, University of Bern, Bremgartenstrasse 109A, 3012 Bern, Switzerland

**Keywords:** miR-128, severe equine asthma, recurrent airway obstruction, microRNA, biomarker, RNA-sequencing, serum, hemolysis, differential expression

## Abstract

MicroRNAs (miRNAs) regulate post-transcriptional gene expression and may be exported from cells via exosomes or in partnership with RNA-binding proteins. MiRNAs in body fluids can act in a hormone-like manner and play important roles in disease initiation and progression. Hence, miRNAs are promising candidates as biomarkers. To identify serum miRNA biomarkers in the equine model of asthma we investigated small RNA derived from the serum of 34 control and 37 asthmatic horses. These samples were used for next generation sequencing, novel miRNA identification and differential miRNA expression analysis. We identified 11 significantly differentially expressed miRNAs between case and control horses: eca-miR-128, eca-miR-744, eca-miR-197, eca-miR-103, eca-miR-107a, eca-miR-30d, eca-miR-140-3p, eca-miR-7, eca-miR-361-3p, eca-miR-148b-3p and eca-miR-215. Pathway enrichment using experimentally validated target genes of the human homologous miRNAs showed a significant enrichment in the regulation of epithelial-to-mesenchymal transition (key player in airway remodeling in asthma) and the phosphatidylinositol (3,4,5)-triphosphate (PIP3) signaling pathway (modulator of CD4^+^ T cell maturation and function). Downregulated miR-128 and miR-744 supports a Th2/Th17 type immune response in severe equine asthma.

## 1. Introduction

Despite intensive efforts, the prevalence of allergies and asthma is still increasing worldwide [1]. Although the therapy for asthma has improved over the years, asthmatic condition can still lead to a sudden death [2,3]. Asthma is a complex disease and hundreds of candidate genes have been proposed for this condition [4,5]. Apart from genetic predisposition, environmental factors seem to play a crucial role in asthma development, including the exposition to indoor and outdoor allergens, such as mites or pollens and irritants like lipopolysaccharides (LPS) [6,7]. Novel techniques and methods, such as next generation sequencing (NGS) have opened a new era in asthma research. Although many genome-wide association studies have been conducted, little replication has been observed [8]. One possible explanation of this phenomenon could be the fact that multiple known phenotypes of human asthma are present and classification depends on clinical phenotypes (severity, treatment-resistance etc.), trigger (allergic/non-allergic asthma, aspirin-induced asthma etc.) and inflammatory phenotype (eosinophilic, neutrophilic, or paucigranulocytic asthma) [9]. Due to the absence of standardized phenotype definitions in the past, interpretation as well as integration of findings proved to be challenging. The most consistently identified candidate genes (interleukin 13 (*IL13),* interleukin 4 (*IL4*)*,* interleukin-4 receptor, alpha (*IL4RA*), cluster of differentiation 14 (*CD14*)*,* Beta-2 adrenergic receptor (*ADRB2*), membrane-spanning 4-domains subfamily A member 2 (*MS4A2/FCER1B*), tumor necrosis factor (*TNF*) superfamily, disintegrin and metalloproteinase domain-containing protein 33 (*ADAM33*), and ORLDM Sphingolipid biosynthesis regulator 3 (*ORMDL3*) do not show association in all of the populations studied or may only show small effects explaining only a very low percentage of the total phenotypic variance [5,10,11].

Severe equine asthma (also called recurrent airway obstruction or heaves) occurs naturally and shares many features with human neutrophilic asthma [12] and also shows parallels to human late-onset and severe asthma [13]. Therefore, asthmatic horses are considered a good animal model for human asthma [13,14]. Asthma in horses has a large economic impact on horse breeding and equestrian sports. Until now, little has been done to prevent the development of asthma in horses. Treatment strategies are focused mostly on a decreased exposure of asthmatic horses to hay, which has been shown to be the major risk factor for asthma development in horses [15,16]. Even though a strong genetic predisposition to severe equine asthma has been reported [17,18,19], excluding affected animals from breeding is difficult. Clinical signs of asthma often appear later than age eight, which is much higher than the average age at which horses are chosen for breeding. Hence, the search for non-invasive biomarkers is of great interest. Potential biomarkers discovered in the equine model could also be further investigated for their implication in human asthma and might even serve as novel therapeutic targets for both equine and human asthma.

Recently, microRNAs in serum (miRNAs) have received great attention as potential biomarkers for many diseases, e.g., neoplastic, cardiac, immune-related, pulmonary and other diseases [20]. MicroRNAs are small RNA molecules that impact biologic responses through the regulation of mRNA transcription and/or translation. A single miRNA may regulate dozens of target genes and thus disrupt an entire genetic pathway leading to pathological features [21].

MicroRNAs are very stable molecules compared to other RNA species and can be transported between cells, tissues and even organisms (mother and fetus) [22]. Extracellular miRNAs can be deregulated in serum and other body fluids during the pathogenesis of many disorders. MicroRNAs from serum are thus of particular interest as promising non-invasive disease biomarkers [23]. Our present understanding of their role in the regulation of allergic diseases is still very limited. However, differential miRNA expression has been shown in a wide range of tissues, cell types, biofluids and vesicles such as bronchoalveolar lavage fluid exosomes, airway T cells and serum from asthmatic patients [24,25]. Since distinct miRNA networks regulate CD4^+^ T cell differentiation, miRNA differential expression studies have the potential to unravel aberrant molecular mechanisms underlying disorders of the immune system [26]. Specifically, miR-155 plays a major role in both allergy and anti-parasitic immunity [27].

Over 1000 miRNAs have been identified in the horse with distinct subsets of miRNAs differentially expressed in a tissue-specific manner [28,29]. Due to their conservation, a majority of equine mature miRNAs have been perfectly matched to human disease-associated miRNAs [30], indicating the potential of investigating miRNA profiles in equine allergic and other conditions [31].

We investigated serum miRNAs and compared the expression profiles of 37 asthmatic Warmblood horses in comparison with 35 unaffected control horses using miRNA-seq. As erythrocyte-derived miRNA may bias the expression profile of serum miRNA [32], we took into account the level of hemolysis in our samples. Furthermore, we retrieved the potential targets of candidate miRNA biomarkers and investigated their expression in peripheral blood mononuclear cells (PBMCs) [33] in correlation with the serum miRNA expression in the same individuals.

## 2. Materials and Methods

### 2.1. Samples

All animal experiments were performed according to the local regulations and with the consent of the horse owners. Sample collection was approved by the Animal Experimentation Committee of the Canton of Bern, Switzerland (BE33/07 (approved 28 March 2007), BE58/10 (approved 19 May 2010), and BE10/13 (approved 19 March 2013)). Phenotyping was performed based on the HOARSI (horse owner assessed respiratory signs index) scoring system [34,35,36]. The HOARSI system is based on the clinical signs and categorizes horses with a score ranging from 1 to 4 (healthy to severe). A HOARSI of 1 indicates no clinical signs of respiratory disease, 2 implies mild clinical signs, 3 means moderate signs and 4 stands for severe clinical signs. Horses presenting a HOARSI of 3 or 4 were considered to suffer from severe equine asthma, while horses with a HOARSI of 1 were used as controls.

We used 79 serum samples derived from Warmblood horses. We reassessed our phenotypic data published before [16,33] and corrected for their status based on reports we received from the horse owners (Table S1). As phenotypic changes for seven horses were reported, these horses were only used for novel miRNA identification but excluded from downstream differential miRNA expression analysis. The age of the control horses ranged from 6 to 32 years (median = 19 years; one horse was six years old, others ≥ 12 years). The asthmatic horses were 9 to 24 years old (median = 16 years) and were in the remission phase of the disease. The studied horses are part of three distinct cohorts: two half-sib families and one unrelated cohort. The blood collection procedure and the investigated individuals were described in our previous studies [16,33,37]. We used 2 mL of serum for the small RNA extraction with the miRNeasy serum/plasma kit (QIAGEN, Hilden, Germany) using an optimized procedure that was described in more detail in a previous publication [38]. In order to verify the level of hemolysis in serum samples, we used a VersaMax ELISA Microplate Reader (Molecular Devices, Sunnyvale, CA, USA) and SoftMax Pro software (version 3.1.2, Molecular Devices). The absorbance in 200 μL of serum was measured at 414 nm, which is the wavelength of maximum absorbance for hemoglobin [32,39]. The quantity of small RNA samples was measured with a QuBit fluorimeter 2.0 (Invitrogen, Carlsbad, CA, USA) and 22 (28%) samples were additionally assessed for miRNA concentration and RNA length distribution with a Bioanalyzer (Agilent, Santa Clara, CA, USA). Next, 5 μL of each small RNA sample was converted into single-end libraries following the standard protocol of the NEBNext Multiplex Small RNA Library Prep Set for Illumina (New England Biolabs, Ipswich, MA, USA). The libraries were then sequenced on five lanes using the Illumina HiSeq 2500 system (Illumina, San Diego, CA, USA) with 50 sequencing cycles.

### 2.2. Data Pre-Processing and Initial Quality Control

Raw sequencing data quality was assessed with FastQC software [40]. Since truncated adapter sequences were reported by FastQC to be over-represented, a FASTA file was generated with the original adapter sequence plus the sequences corresponding to truncated adapters reported by FastQC for every sample. These multiple adapter sequences, as well as low quality base calls (q < 20), were trimmed with cutadapt (v. 1.8) [41] with the following options: --trim-n -a file:‘sample specific adapter sequences in FASTA file’ -m 15 -q 20. The resulting read length distribution was further analyzed as next quality control step. The sequencing dataset of 42 controls and 37 cases is available at the European Nucleotide Archive (ENA) [42].

### 2.3. Novel miRNA Identification

For the identification of potential novel equine miRNAs, the quality and adapter trimmed reads were mapped to the reference genome (EquCab2.0) [43] using miRDeep2 (v.0.0.7) [44]. Briefly, miRDeep2 predicts novel miRNAs by aligning the reads to a reference genome and extracting candidate pre-miRNA sequences from the aligned genomic DNA loci and these extracted pre-miRNA sequences are assigned a score based on the ability of the precursor to fold to a pre-miRNA like secondary hairpin structure, the absolute and relative number of reads mapping to the three distinct precursor products that result from dicer processing (5p arm, 3p arm and loop), the possible conservation of the 5′ end and the presence of a 3′ overhang in the mature sequence [45]. If the algorithm fails to identify a stem-loop product within the candidate, then the miRNA precursor is rejected. The processed reads from all 79 FASTQ files were mapped using the mapper.pl script of the miRDeep2 tool in one run using a configuration file containing a list of all FASTQ files names and a three-letter code for each sample. The module maps the reads to the genome with Bowtie1 [46] keeping only the alignments with 0 mismatches (option -n) in the seed region and only reads that do not map to more than five different loci in the genome are kept (option -m). The module was run with the following parameters: -d -v -u -e -h -o 16 -m -n -q -p. Subsequently, the core algorithm module miRDeep2.pl was used to discover potential novel miRNAs as described previously [29]. MiRDeep2 assigns each novel miRNA a log-odds score (further referenced as miRDeep2 score) that represents the probability that the precursor is a true miRNA precursor based on the theory of miRNA processing by dicer as well as the actual data and the alignment pattern of the reads. Any pre-miRNA candidate with a miRDeep2 score of at least 0 was considered as a potential (predicted) miRNA. To filter for predicted potential novel horse-specific miRNAs with high confidence, a miRDeep2 score cut-off of four was adopted. This cut-off was set based on our initial analysis: the signal-to-noise ratio (total miRNA precursors reported divided by the mean estimated false positive miRNA precursors over 100 rounds of permuted controls) reached a reasonable level of 9.4:1 at that threshold while the value dropped drastically for lower miRDeep2 scores. Additionally, the percentage of the detected novel miRNAs that were estimated to be true positives dropped drastically too at that threshold (82% true positive rate at a miRDeep2 score of 4 versus a rate of 54% true positive rate at a miRDeep2 score of 3; Table S2). Human known miRNAs were used as a guide for the novel equine miRNA identification. Known equine and human miRNAs were obtained from the miRBase database, release 21 [47]. Finally, overlapping novel miRNAs (by at most 1 nucleotide) were merged using BED tools [48].

### 2.4. Identification of a Read Count Threshold to Select Relevant miRNAs

The distribution of miRNA transcript counts in our dataset showed three distinct phases, similar to that described in Koh et al. [49]. The first phase with low count miRNAs likely represents noise in the dataset, due to random expression of these transcripts. The second phase shows similar expression between replicates which may be attributed to steady state expression or consistently expressed miRNA transcripts. Finally, the last phase consists of miRNAs with large count numbers. Kolmogorov-Smirnov test (KS-test) was used to examine the similarity of the empirical distributions between replicates and distinguish biologically relevant miRNAs from the noisy transcripts. The *D* statistic of a given KS-test near zero indicates similar distributions, while a larger test statistic indicates more severe bias. Similar to Koh et al. we used the *D* statistic as a cost function, hence we first determined the *D* statistic of the whole dataset and then determined the *D* statistic by iteratively excluding miRNAs not achieving a required minimal read count level [49]. As we changed the minimum threshold value for exclusion of low count miRNAs, we arrive at a low *D* statistic point indicating similar distributions. The *D* statistic point where this first occurs is designated as the minimum threshold count value of biological significance (code available on GitHub [50]).

### 2.5. Differential Expression Analysis

The dataset used for differential expression analysis consisted of a total of 71 horses: 34 controls (19 females and 15 males) and 37 affected horses with severe equine asthma (one principal component analysis (PCA) outlier and 7 phenotypically changed horses were excluded) (Table S7).

For differential expression analysis, both novel and known equine miRNAs (miRBase v21) were used. The quantification of miRNA expression level was carried out using the quantifier.pl script of the miRDeep2 tool applying the parameters -k -j.

MicroRNAs not meeting the threshold for biological relevance determined by the KS-test approach were filtered out. Furthermore, we removed lowly expressed miRNAs (showing zero counts in more than 10% of samples). The filtered miRNAs were then quality controlled using PCA (top 50 variable miRNAs) based clustering. The PCA plot was produced using variance-stabilized counts [51]. Differential expression analyses were carried out with edgeR (v. 3.16.5) [52] and DESeq2 (v. 1.14.1) [53]. For DESeq2, we used the DESeq function, which estimates size factors and dispersions and finally fits a model in order to perform differential expression tests using negative binomial generalized linear models. For edgeR, the function calcNormFactors was used to normalize the count data, while the function estimateDisp was used to estimate the dispersions and likelihood ratio test for generalized linear models (glmLRT) for the differential expression tests. For both tools, the linear model applied corrected for the levels of hemolysis as well as the underlying population structure (Table S1). MicroRNAs with a false discovery rate (FDR) adjusted *p*-value (adjusted *p*) determined by DESeq2 below our FDR threshold of 0.05 were considered as differentially expressed (up/downregulated) and will further be referenced as differentially expressed miRNAs (DEmiRs).

### 2.6. mRNA-miRNA Interaction

We next retrieved potential target genes of the DEmiR, showing the lowest adjusted *p*-value, eca-miR-128, with TargetScan (v. 6.2) [54]. Human gene symbols of the target genes were converted to horse Ensembl IDs with the Ensembl biomart tool [55]. TargetScan searches for conserved target-sites in the 3′ UTR of equine genes. The human homologous gene symbols of the potential target genes were used for Gene Ontology (GO) (GO, release 106) biological process network enrichment analysis with the GeneCodis tool [56,57,58,59]. The list of reported target genes was intersected with a list of previously published differentially expressed mRNA in PBMCs of the same horses in order to investigate potential regulatory miRNA-mRNA networks [33].

### 2.7. Functional Enrichment of DEmiR Target Genes

A high confidence set of experimentally-validated target genes for all DEmiR (human homologues) was obtained from DIANA-TarBase v. 7.0 [60] using a stringent prediction score threshold of 0.9 (Table S8). The resulting set of high-confidence target genes was used with the GeneGo database to perform a functional enrichment analysis. MetaCoreTM v. 6.32 (Thomson Reuters, London, UK) software was used for GeneGo pathway and network analysis. The auto expand algorithm was used for building a functional network.

### 2.8. Code

The scripts that were used for the analyses of this study can be found on GitHub [50].

## 3. Results

### 3.1. Small RNA Sequencing and Data Quality Control

**Figure 1:**
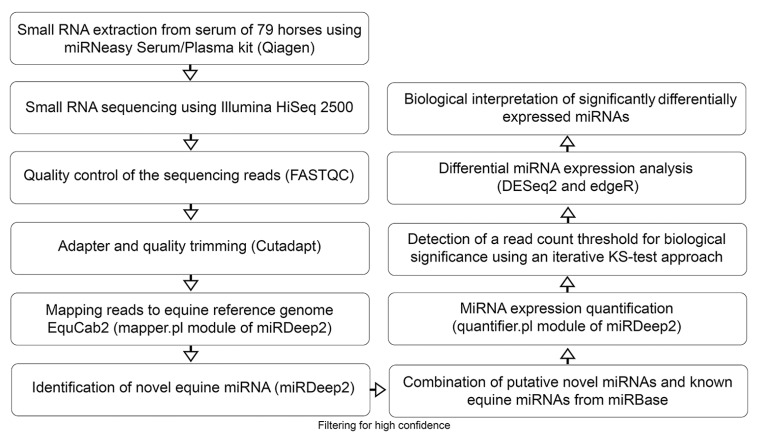
Flow chart outlining the pipeline for novel miRNA detection as well as miRNA expression profiling and differential expression analysis. After extracting small RNA from serum samples of 79 horses, the samples were sequenced and analyzed using state of the art bioinformatics tools. While all samples were used for the novel miRNA identification, only 71 horses were used for the differential miRNA expression analysis.

As a first step, small RNAs were extracted from 79 horse serum samples (Figure 1). Few samples were visibly hemolytic and the absorbance values at 414 nm ranged from 0.53 to 4 (median = 1). We used between 12.55 ng and 88.00 ng (mean = 36.60 ng) of the small RNA extracts for the library preparation. Subsequent to an initial quality control step using FASTQC [40], we performed adapter and quality trimming. The mean percentage of total reads remaining after applying cutadapt [41] was 86.6% and truncated adapters were trimmed from an average of 0.34% of total reads. After pre-processing of the raw reads, we obtained 11.7 million reads per library on average. The mean number of reads mapped by Bowtie1 to the horse reference genome per library was 6.99 million.

**Figure 2:**
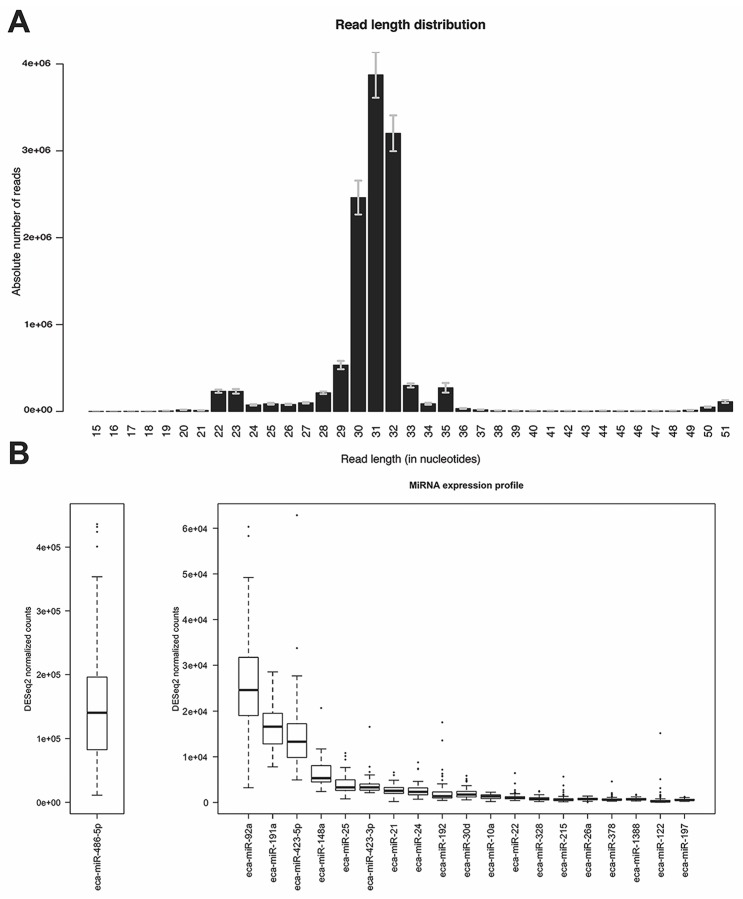
Read length distribution and miRNA expression profile. (**A**) Read length distribution after adapter and quality trimming for all 79 samples. The error bars indicate the standard error of the mean; (**B**) Boxplots of the DESeq2 normalized read counts of the 20 most highly expressed known miRNAs in all samples. For better representation of the data, the top expressed miRNA eca-miR-486-5p is shown in a separate plot with a different scale on the y-axis.

Analysis of the read length distribution of all samples revealed a clear bimodality where on average 4.6% of the total reads constituted the first peak between 21 and 24 nucleotides (nts) while the second and more prominent peak was located between 29 and 33 nts with 95% of the total reads falling into this range (Figure 2A). Since the percentage of mapped reads to the equine genome showed a rather low value of median 38% using Bowtie1 [46] (mapper.pl module), different mapping algorithms were applied to investigate the impact on the percentage of mapped reads and the impact on the percentage of reads mapped to annotated regions coding for miRNAs. Even though the percentage of reads mapped to the equine genome was increased to a median of 70% for Burrows-Wheeler Aligner (BWA) [61] (parameters -n 1 -o O -e O -k 0 -l 8 -t 4) and 95% for Bowtie2 [62] (parameters -q –very-sensitive-local), the percentage of reads mapping to known miRNA regions relative to the number of total reads was higher in case of Bowtie1 (4% of reads mapped to known miRNAs) when compared to Bowtie2 and BWA (2% mapped to known miRNAs). Thus, the mapper.pl script (implementing Bowtie1) was used to map the small RNA sequencing reads to the equine genome. After mapping, the reads were collapsed into 303,000 unique reads per library on average and were used for novel miRNA identification and expression quantification with miRDeep2 [44].

### 3.2. Novel miRNA Identification

The core algorithm of miRDeep2 reported a total of 721 putative novel miRNAs with a miRDeep2 score between 0 and 10 (Table S2). After applying our miRDeep2 score cut-off of 4 to filter for high confidence novel miRNAs, a set of 192 novel miRNAs remained. Of this subset of potential novel miRNAs, three mapped to ribosomal or transfer RNA regions (Rfam v. 12.2) [63] and showed non-significant randfold *p*-value suggesting the secondary structure was unlikely to match the one of a miRNA precursor. This resulting set of high confidence novel miRNAs is listed in Tables S3 and S4. The precursors of 47 of the novel miRNAs overlapped with a set of novel equine miRNA precursors identified in a previous study of our group [33].

### 3.3. Threshold Read Count Value Determination by KS-Test

A threshold value of 13 (DESeq2 mean normalized) read counts was set for biological significance by applying the KS-test approach (see Materials and Methods for details). Therefore, the base mean counts for expressed miRNAs had to be ≥13 to be considered biologically relevant.

### 3.4. MiRNA Expression Profile

**Figure A1:**
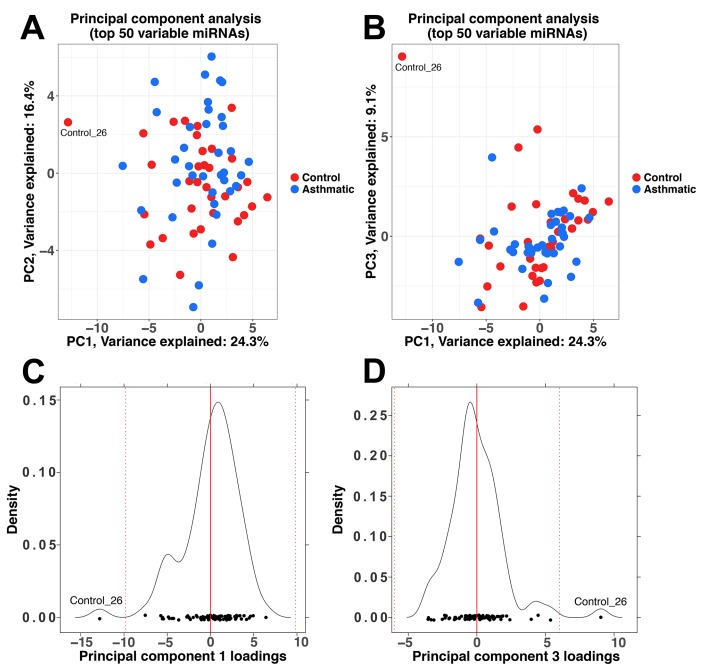
Principal component analysis (PCA) including the 50 most variable miRNAs. (**A**) PCA plot of principal component (PC) 1 versus PC2; (**B**) PCA plot of PC1 versus PC3; (**C**) Density plot of the PC1 loadings. The red line indicates the mean of the values, while the dotted red lines represent the mean value ± three standard deviations; (**D**) Density plot of the PC3 loadings.

We detected 515 miRNAs (189 novel predicted miRNAs and 326 known miRNAs) to be expressed in our dataset. After filtering for low counts and mean threshold values of ≥13 normalized counts across all samples, 91 miRNAs remained, which were later used for differential expression analysis. Of the filtered miRNAs 61 (67%) were expressed at a low to moderate level (10–250 DESeq2 normalized counts on average), 16 miRNAs (18%) were expressed at a moderate to high level (251 to 999 DESeq2 normalized counts) while 14 miRNAs (15%) showed high expression levels of more than 1000 DESeq2 normalized counts. Of the total counts 86% were contributed by the top five most highly expressed miRNAs (eca-miR-486-5p, eca-miR-92a, eca-miR-191a, eca-miR-423-5p, eca-miR-148a). The miRNA with the highest expression counts was eca-miR-486-5p showing 63% of the total counts (Figure 2B). A PCA plot was generated with the 50 most variable miRNAs. Even though no clear clusters of case and control horses were formed, one outlier sample was detected and removed. This outlier was more than three standard deviations away from the mean of the respective principal component loadings on principal component 1 and 3 and was therefore excluded from further analyses (Figure A1). Thus, the resulting dataset for the following downstream analyses consisted of 71 samples.

### 3.5. Hemolysis Effect on miRNA Expression Profile

**Figure A2:**
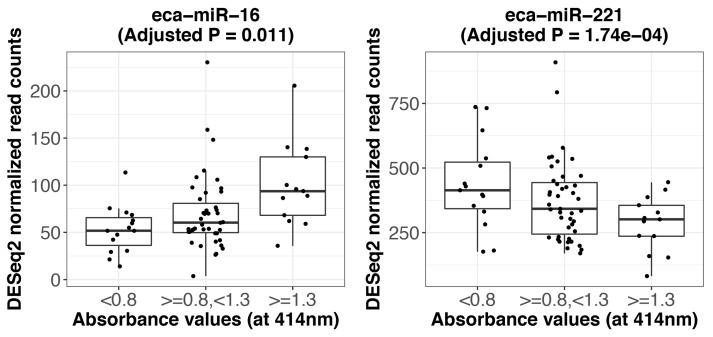
Expression of miRNAs significantly affected by the level of hemolysis. The expression (DESeq2 normalized read counts) of eca-miR-16 shows a significant positive association with increasing level of hemolysis (adjusted *p* = 0.011, log2 fold change = 0.44) while the expression level of eca-miR-221 is negatively affected by an increasing level of hemolysis (adjusted *p* = 1.74 × 10^−4^, log2 fold change = −0.54). For visualization purposes, the samples were grouped into three groups according to the hemolysis levels: absorbance at 414 nm < 0.8 (*n* = 15), absorbance ≥ 0.8 and <1.3 (*n* = 44) and absorbance ≥ 1.3 (*n* = 13).

Twenty-six miRNAs were significantly affected by the level of hemolysis at a FDR threshold of 0.05 according to DESeq2 (Table S5). Figure A2 depicts two miRNAs representing expression levels of miRNAs that are either positively or negatively affected by the level of hemolysis. The analysis with the tool edgeR showed following differenced in contrast to the analysis with DESeq2: four miRNAs were not significantly affected by the level of hemolysis (eca-miR-744, eca-miR-128, eca-miR-28-3p and eca-miR-125a-5p) and additionally five significantly affected miRNAs were reported: eca-miR-423-5p, eca-let-7g, eca-miR-19b, eca-miR-425, eca-miR-7177b (Table S6). Hence the following linear model was used to determine differentially expressed miRNAs between asthmatic and control horses which accounts for the hemolysis effect and the underlying population structure:

~ absorbance values+family1+family2+condition

The two binary covariates *family1* and *family2* encode the underlying population structure of three cohorts: *family1*, *family2* and an unrelated cohort.

### 3.6. Asthma Related miRNAs

**Figure A3:**
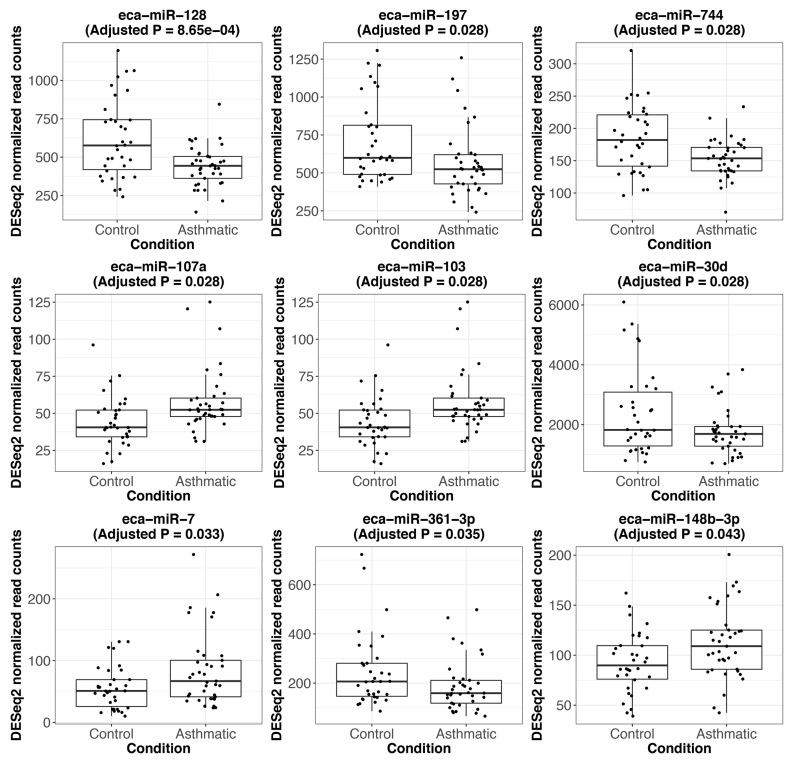
Expression of significantly differentially expressed miRNAs between asthmatic and control horses. Boxplots showing the DESeq2 normalized read counts for the statistically significant DEmiRs. The respective FDR-adjusted *p*-value (adjusted *p*) is indicated.

Using DESeq2, we identified 11 miRNAs as statistically significant DEmiRs after accounting for the level of hemolysis: eca-miR-128, eca-miR-744, eca-miR-197, eca-miR-103 and the closely related eca-miR-107a, eca-miR-30d, eca-miR-140-3p, eca-miR-7, eca-miR-361-3p, eca-miR-148b-3p and eca-miR-215. Eight of these eleven DEmiRs were also reported by edgeR (eca-miR-7, eca-miR-148b-3p and eca-miR-215 missed the significance threshold) (Table 1). The log2 fold changes of statistically significant DEmiRs varied between −0.49 and 0.47 (Figure A3).

### 3.7. mRNA-miRNA Interactions

**Figure 3:**
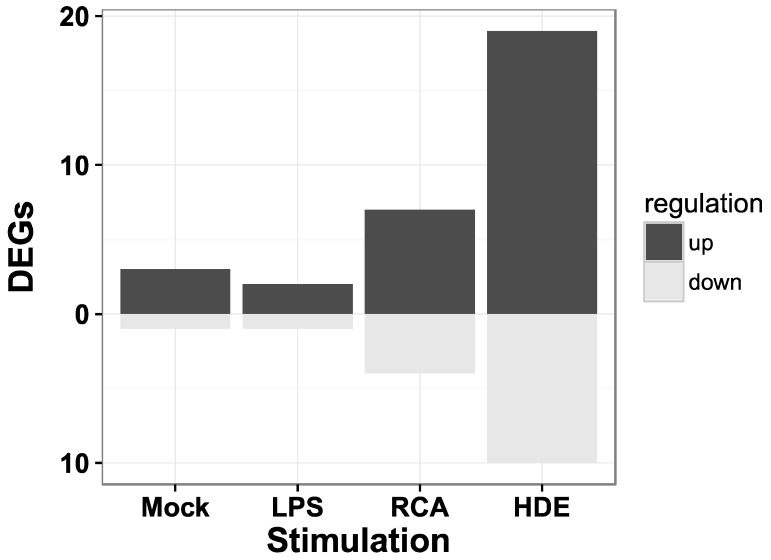
The number of miR-128 target genes identified as differentially expressed genes (DEGs) in peripheral blood mononuclear cells (PBMCs). The list of target genes of miR-128 was intersected with the list of DEGs from a previous study analyzing mRNA expression in PBMCs. These PBMCs were derived from the same blood samples as the serum analyzed in this study. The PBMCs were either unstimulated (Mock) or stimulated with lipopolysaccharides (LPS), recombinant cyathostomin antigen (RCA), or hay dust extract (HDE) [33].

We retrieved 212 potential (predicted and experimentally known) target genes of the miRNA with the lowest adjusted *p*-value, eca-miR-128 from the TargetScan database. The most significantly enriched GO biological processes (release 106) were signal transduction (GO:0007165, corrected hypergeometric *p*-value, Hyp* = 4.33 × 10^−4^) and regulation of transcription, DNA-dependent (GO:0006355, Hyp* = 4.47 × 10^−4^) [56,57,58,59]. Of the 212 human target genes 174 genes (82%) are annotated in the horse and 30 of them were previously reported to be differentially expressed in PBMCs of asthmatic horses when stimulated with either Mock, LPS, recombinant cyathostomin antigen or with hay dust extract in comparison to control horses (Figure 3) [31].

### 3.8. Functional Enrichment of DEmiR Target Genes

For investigating functional enrichment of DEmiR target genes, we first constructed a high-confidence set of experimentally validated target genes of all the DEmiRs using the DIANA-TarBase database v. 7.0. This resulted in a set of 576 unique target genes. Table S8 illustrates all the target genes for each DEmiR. The top 10 most enriched GeneGO pathway maps associated with the 576 target genes regulated by DEmiRs are shown in Table 2.

Biological process networks in the GeneGo database analysis are networks of cellular functions and regulations based on functionally interconnected pathways. Process network analysis showed statistically significant enrichment for developmental networks (hedgehog signaling, epithelial-to-mesenchymal transition), signal transduction (NOTCH signaling, WNT signaling), translation (regulation of initiation), cell cycle (G1-S growth factor regulation, G1-S interleukin regulation, G1-S), cell adhesion (amyloid proteins) and proliferation (positive regulation cell proliferation).

**Figure 4:**
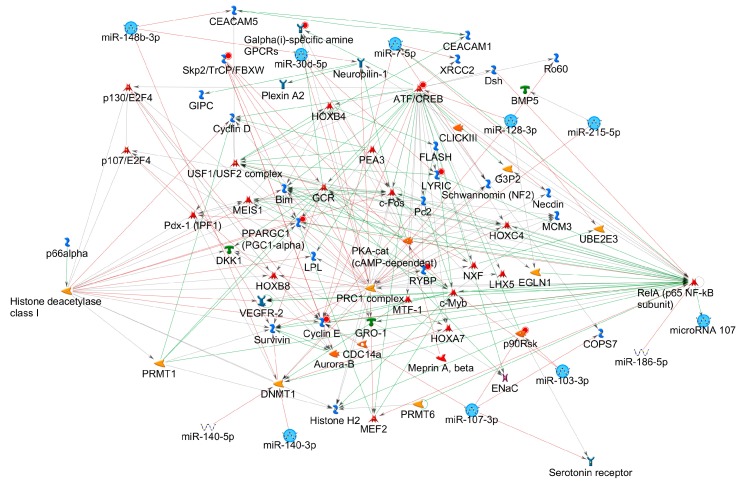
miRNA-protein network. Network built using MetaCore (Thomson Reuters) including nine interconnected significant DEmiRs (light blue circles).

The enrichment for diseases resulted in an enrichment of respiratory tract related diseases (lung neoplasms, respiratory tract neoplasms, thoracic neoplasms, respiratory tract diseases, lung diseases, rectal neoplasms, genital neoplasms female, rectal disease, glioma, neoplasm neuroepithelial). We then investigated the regulatory miRNA-protein interaction networks using GeneGo database with homologous human miRNAs. This analysis showed nine of the eleven significant DEmiRs are part of an interconnected network containing the following highly interconnected hubs: ATF/CREB (activating transcription factor/cAMP response element modulator) family, polycomb repressive complex 1 (PRC1), Cyclin E, c-Fos and RelA (p65 NF-κB subunit) and histone deacetylase class I (Figure 4).

## 4. Discussion

In the light of the constantly increasing number of allergies in humans [1] and also among companion animals [64], there is an urgent need for non-invasive biomarkers to help predict the risk of asthma development. We used a large cohort of 71 mature horses of different age, sex and country of origin, to search for a non-invasive biomarker for asthmatic condition and a better understanding of the pathology of the disease. Using the tool miRDeep2, we were able to identify 142 putative novel equine miRNAs in serum. The high-confidence novel miRNAs were combined with the known equine miRNAs from miRBase and expression profiling followed by differential expression analysis was performed. In agreement with previous reports of plasma expressed miRNAs in horses, one of the top expressed miRNAs across all samples was eca-miR-486-5p [65].

### 4.1. Hemolysis Effect on miRNA Expression Profile

MiRNAs that are affected by the level of hemolysis have been shown to be limited in their applicability as disease biomarkers [32,66] and in line with these results, we report 26 miRNAs with expression levels that are affected by the level of hemolysis (analysis performed with DESeq2). MiR-486 is known to be highly expressed in blood cells, mainly erythroid cells [67,68] and was one of the 26 miRNAs significantly affected by the level of hemolysis. From the four miRNAs commonly reported as showing a hemolysis-affected expression (miR-451, miR-16, miR-92a and miR-486p) [32,39,66] only eca-miR-486-5p (adjusted *p* = 2.5 × 10^−4^) and eca-miR-16 (adjusted *p* = 0.011) showed a fold-change affected by the level of hemolysis. Some miRNAs show a decreased expression level with higher levels of hemolysis which can possibly be explained by increased presence of nucleases or other destabilizing agents released from lysed erythrocytes influencing miRNA turnover and thus leading to the degradation of certain miRNAs [69]. We accounted for hemolysis in our linear model, however lysis of other cell types could impact the results.

### 4.2. Asthma Related miRNAs

**Figure A4:**
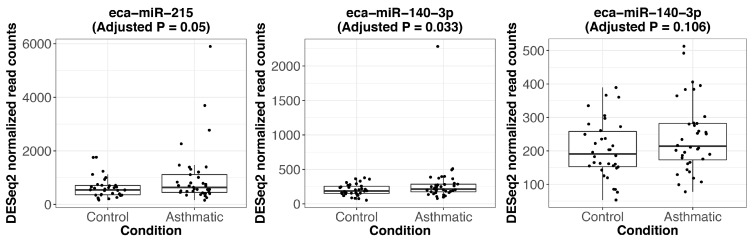
Expression of significantly differentially expressed miRNAs between asthmatic and control horses. Boxplots showing the DESeq2 normalized read counts for the significant DEmiRs. The respective FDR-adjusted *p*-value (adjusted *p*) is indicated. The first plot indicates the borderline significant eca-miR-215 followed by the boxplot for eca-miR-140-3p which clearly indicates that the signal for that miRNA is predominantly driven by one extreme outlier individual. After the exclusion of that outlier, eca-miR-140-3p did not show significant differential expression anymore (last plot; adjusted *p* = 0.106).

Differential miRNA expression analysis between asthmatic and control horses identified eleven statistically significant miRNAs (Table 1). The differential expression of eca-miR-140-3p is considered to be of low confidence, since the signal was mainly driven by just one sample and the differential expression was not significant after the exclusion of the outlier individual (Figure A4). However, since the specific outlier individual was not a global outlier (as shown by PCA) but rather only for this one miRNA, this individual was still kept for differential expression analysis. Thus, the remaining ten high-confidence DEmiRs between asthmatic and control horses were used for downstream literature research to investigate their possible biological involvement in the pathophysiology of severe equine asthma.

### 4.3. Biological Implications: Th2/Th17/Th1 Immune Response

The top differentially expressed miRNA (miR-128) has already been implicated in carcinomas [70,71] and serum miR-128 was suggested as potential biomarker for e.g., glioma [72]. A potential role of miR-128 in horse asthma may result from its pro-apoptotic properties [73,74]. This hypothesis is further supported by previous studies on mRNA in asthmatic horses that showed disturbances in the cell cycle [33,75,76,77].

MiR-128 has recently been shown to be downregulated in asthmatic bronchial epithelial cells and to be part of a regulatory miRNA network that was confirmed to significantly increase the production of interleukin 6 (IL6) and interleukin 8 (IL8) [78]. The increased presence of the pro-inflammatory IL6 and IL8 were previously shown to be associated with the pathophysiology of asthma [79,80]. IL8 is a chemokine that is responsible for the recruitment and activation of neutrophil granulocytes and multiple studies showed its overexpression in bronchoalveolar lavage cells and airway epithelium of horses with severe equine asthma [81,82,83]. Consequently, airway neutrophilia is a prominent feature shown by horses suffering from severe equine asthma [13]. The pleiotropic cytokine IL6 is viewed as a marker of airway inflammation in asthma (showed in humans and animal models) and has been proposed as a therapeutic target in clinical trials [84,85].

The observed differences in the levels of several cytokines in equine asthma can most likely be explained by alterations in the CD4^+^ T cell development pathway. Downregulated miR-128 has been shown to positively regulate CD4^+^ T cell differentiation to T helper 2 (Th2) cells and to negatively regulate Th1 cell maturation. Increased levels of IL6 as well as TGFβ are known to positively regulate T cell maturation to T helper 17 (Th17) cells [26]. The differentially expressed miR-744 was shown to target *TGFβ*, thus its downregulation in horses suffering from severe asthma, as shown in this study, likely leads to increased levels of TGFβ [86].

Furthermore, increased levels of interleukin 17 (IL17A) in bronchoalveolar lavage cells of horses suffering from severe equine asthma were reported, suggest the increased presence of Th17 cells [87]. It is known that Th17 cytokines (IL17A, interleukin 17F (IL17F), and interleukin 22 (IL22)) lead to mucous cell metaplasia as well as increased levels of airway remodeling [88]. Also, differentially regulated miR-197 was shown to be involved in the reciprocal regulation of the *IL6/STAT3* pathway [89]. Interestingly, the STAT3 transcription factor is essential for Th17 cell differentiation [90,91].

It has previously been stated that an exaggerated Th2 response as well as a Th17 response is able to explain a large portion of the pathophysiological events underlying severe equine asthma [13].

An enhanced Th17 response is also supported by a finding in a previous RNA sequencing study investigating the transcriptome of unstimulated and stimulated PBMCs collected from the same horses that this study covers. We showed a significant increase in C-X-C motif chemokine ligand 13 (*CXCL13)* transcript abundance as well as a decrease in interferon gamma (*IFNG*) expression in horses affected by severe equine asthma when compared to control horses [33]. CXCL13 is a B cell attracting chemokine that was shown to be predominantly produced by Th17 cells but not Th1 or Th2 cells [92]. Increased CXCL13 expression was linked with the formation of ectopic lymphoid structures like inducible bronchus-associated lymphoid tissue (iBALT) [93]. The formation of iBALT was recently shown to be dependent on Th2 as well as Th17 immunity in the course of a fungal infection of the lung in mice [93]. Additionally, iBALT was shown to be present in 90–100% of human asthmatic individuals and the abundance is correlating with asthma severity [94]. On the other hand IFNG is known to be the hallmark cytokine of Th1 cells and its downregulation further supports a decline of Th1 cell abundance [95].

An alternative hypothesis is that horses with severe asthma show dual positive Th2/Th17 cells which were recently discovered and were already reported to be present in an elevated number in the BAL fluid of individuals with severe asthma [96]. These striking concordances with this study further strengthen the hypothesis of a predominant Th2 and Th17 immune response in severe equine asthma.

**Figure 5:**
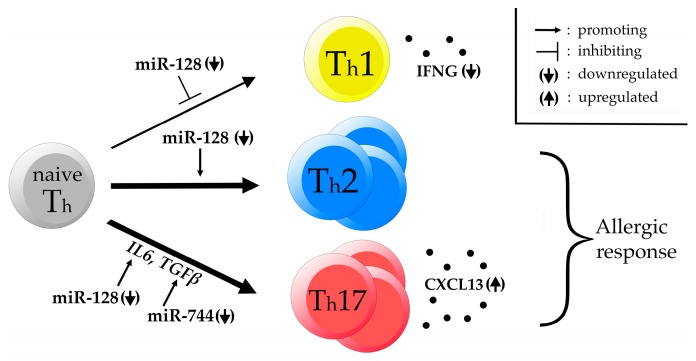
DEmiRs affecting CD4^+^ T cell development. Hypothetical network of DEmiRs affecting T helper (Th) cell maturation. Downregulated miR-128 is known to promote Th cell development towards the Th2 side while negatively regulating Th1 cell maturation. Additionally, IL6 production is increased by downregulated miR-128 while TGFβ production is enhanced by downregulated miR-744. We hypothesize that this leads to an increased Th2/Th17 immune response upon antigen challenge rendering certain horses susceptible to develop severe equine asthma. This hypothesis is supported by a previous finding of downregulated interferon gamma (IFNG) and upregulated C-X-C motif chemokine ligand 13 (CXCL13) in PBMCs of the same affected horses.

Accordingly, we also hypothesize that the significant deregulation of the ten miRNAs, as shown in this study, might be a factor influencing the susceptibility of certain individuals to develop asthma due to a deregulation in the T cell maturation pathway leading to polarization of the immune response towards the Th2 and Th17 side and away from the Th1 side (Figure 5).

Rather than focusing on anti-IL6 agents to control the IL6 pathway (or other cytokines) as a therapeutic approach for asthma and other disease as it has recently been proposed, a valid novel approach could therefore be to consider the reported DEmiRs as potential therapeutic targets. Following this approach, it might be able to prevent an amplified Th2/Th17 response and shift the balance back to an increased Th1 cell differentiation [84].

### 4.4. Biological Implications: Asthma and Cell Cycle Regulators

The closely related miRNAs miR-103 and miR-107a are known to pilot cell cycle arrest and their up-regulation in horses suffering from severe equine asthma support previous findings about cell cycle disturbances in equine asthma [33,97,98]. Compelling accordance with our results was provided by a recent study covering the autoimmune disease lupus erythematosus, a chronic inflammatory condition [99]. This study reported a deregulation of the cell cycle characterized by an upregulation of various miR-15/16 members, including miR-103 and miR-107a, as well as a downregulation of miR-744. Hence, upregulation of miR-103 and miR-107 as well as downregulation of miR-744 in asthmatic horses highlight a potential role of this miRNA network in chronic inflammatory conditions.

On the other hand, the significantly differentially expressed miR-361-3p is known to act as a tumor suppressor by interfering with the cell cycle. Interestingly this miRNA was recently shown to be significantly downregulated in patients affected by Sézary Syndrome, an aggressive CD4^+^ T-cell lymphoma [100]. Thus, the downregulation of eca-miR-361-3p in severe equine asthma might lead to the increased proliferation of CD4^+^ T-cells, or it might exhibit a yet unknown function in CD4^+^ T-cell differentiation.

### 4.5. Known Biological Implications of the Remaining DEmiRs

The epidermal growth factor receptor (EGFR)-mediated miRNA miR-7 is a known key player in multiple lung-related diseases and has previously been proposed as a biomarker in serum for chronic obstructive pulmonary disease and is thought to act by suppressing the coupling of SWI/SNF-related matrix-associated actin-dependent regulator of chromatin subfamily D member 1 (SMARCD1) with p53 [101,102]. MiR-148b-3p inhibits the expression of major histocompatibility complex (class I, G; HLA-G). *HLA-G* is a known susceptibility gene for asthma and therefore it has previously been proposed that miR-148b-3p might overtake a role in asthma susceptibility by interacting with *HLA-G* [103,104]. The differentially expressed miR-215 has been shown to target *IL17RS* and *IL21* [105,106]. Finally, miR-30d has previously been proposed as biomarker for asthma severity and is known to lead to airway smooth muscle hypertrophy [66,107].

Since all 11 DEmiRs detected in the course of this study showed a small log2 fold change between case and control horses, these miRNAs might not act as a non-invasive diagnostic biomarker for equine asthma due to the low specificity of a potential test. However, the DEmiRs of this study still provide novel insights about the possible underlying pathophysiology of severe equine asthma and thus highlight candidate pathways to target in future therapeutic approaches.

### 4.6. mRNA-miRNA Interactions

For further in-silico downstream analysis we focused on the most significant DEmiR eca-miR-128. Potential target genes of miR-128 are involved in signal transduction, an indisputable part of immune response. Signal transduction was also the most enriched biological process by the differentially expressed Genes (DEGs) related to asthma in equine PBMCs [33].

All three up-regulated DEGs (RAB20, member RAS oncogene family (*RAB20*)*,* bromodomain adjacent to zinc finger domain 2B (*BAZ2B*), H2.0 like homeobox (*HLX*)) in mock stimulated control and asthmatic PBMCs derived from the same blood sample collection as the serum samples used in this study are potential targets of miR-128 and were previously implicated in human asthma-like diseases [108,109]. A single nucleotide polymorphism (SNP) in *RAB20* has been associated with childhood asthma in European-American and Hispanic-American populations using genome wide association study (GWAS) [110]. Using the same technique, an SNP within *BAZ2B* was associated with longitudinal changes in lung function and mean rates of decline by smoking pattern [111,112]. However, little is known about the *BAZ2B* function.

The third gene, *HLX*, is a Th1 specific transcription factor (TF) that interacts with another Th1 specific TF, T-box 21 (*TBX21*) required for the Th1 cells maturation and Th1-specific cytokine expression, including high expression of *IFNG* and repression of *IL4* expression [113,114,115]. Variation in the genes of both the TFs have been associated with childhood asthma and variation within *HLX* significantly decreased its activity [116,117,118]. It is likely that an increased expression of *HLX* in asthmatic horses is maintained due to the decreased activity of the TF enforced by silencing of the *HLX* transcript by miR-128. Whereas childhood asthma is thought to be a Th2-related type of asthma [119], severe asthma in humans is characterized by high levels of Th1 cells [120]. Therefore, the decreased expression of miR-128 and increased expression of its target, the *HLX* serve as potential therapeutic targets for equine as well as human asthma.

### 4.7. Functional Enrichment of DEmiR Target Genes and Network Analysis

Functional pathway enrichment using MetaCore (Table 2) revealed that the most significantly enriched pathway map was the regulation of epithelial-to-mesenchymal transition (EMT) (adjusted *p* = 1.02 × 10^−4^). EMT has been proposed to be a key player in airway remodeling in asthma [121,122]. The second most significant pathway map, PIP3 signaling, takes over a prominent function in airway inflammation. PIP3 is produced by PI3K and leads to the stimulation of several downstream targets including the Akt kinase. The PI3K pathway overtakes a major role in CD4^+^ T cell differentiation and activation, supporting our hypothesis of an altered CD4^+^ T cell development in severe equine asthma [123]. Blocking the PI3K/Akt signaling pathway has previously been proposed as a therapeutic approach for early stages of airway remodeling induced by the abnormal epithelial-to-mesenchymal transition [124].

Phosphorylation of EIF2 function pathway (adjusted *p* = 4.29 × 10^−4^) is required for the activation of NF-κB which in turn leads to increased lung inflammation in response to an allergen challenge [125]. The regulation of GSK3β functional pathway (adjusted *p* = 0.29 × 10^−4^) is essential for regulatory T cell function and has been proposed as a therapeutic target against allergic airway inflammation [126,127].

The biological process networks enrichment analysis resulted in a significant enrichment in the NOTCH signaling pathway, which is of special interest, since it was shown that the NOTCH pathway is crucial for Th17 cell differentiation [128].

The miRNA-gene network analysis revealed multiple hubs like ATF/CREB, PRC1, Cyclin E, c-Fos and RelA (p65 Nuclear Factor-κB subunit) and histone deacetylase class I (Figure 4).

Several of these most prominent hubs were previously implicated in asthma pathophysiology. The autoimmune disease modulating ATF/CREB pathway has been shown to promote Th17 differentiation together with CRTC2 [129]. Increased c-Fos expression in T lymphocytes has been shown to be involved in corticosteroid-resistant bronchial asthma [130]. The well-characterized NF-κB is a key regulator of adaptive and innate immune response that plays a pivotal role in allergic airway diseases [131]. Besides, the histone deacetylase class I hub confirms recent findings in human and murine model asthma research where histone deacetylases were reported to play an important role in asthma pathogenesis and histone deacetylate inhibitors showed promising results in asthma treatment studies in animal models [132]. PRC1 is responsible for gene silencing by post-translational modification of histones and exerts important functions in T cell differentiation. PRC1 recognizes H3K27me3 epigenetic modifications and condenses chromatin, thus leading to stabilized Th2 cell function and restricting the plasticity of the cells towards the Th1 side [133].

## 5. Conclusions

Using small RNA sequencing data from 71 individual horses, we identified 11 significantly differentially expressed miRNAs in the serum of asthmatic horses compared to controls. Several of the DEmiRs have previously been implicated in cell cycle control and some of them are known modulators of CD4^+^ cell differentiation and airway remodeling. This confirms a previous finding of our group reporting an impaired cell cycle control in RAO horses. We hypothesize that the immune response underlying the pathophysiology of severe equine asthma follows a Th2/Th17 driven manner rather than one of type Th1. This is backed by the fact that downregulated miR-128 was shown to negatively regulate T cell maturation towards Th1 but to also positively regulate the maturation towards Th2 cells. Additionally, a modulated cytokine profile towards the IL6 and TGFβ side caused by decreased levels of miR-128 and miR-197, as well as increased levels of miR-744 positively affect the maturation of T cells towards the Th17 side.

Therefore, we propose that the decreased levels of serum miR-128 might yield insights into the molecular mechanisms underlying asthma and might also provide room for novel therapeutic strategies. Rather than focusing on anti-interleukin agents for therapeutic approaches we propose that the identified differentially expressed miRNAs might act as novel therapeutic target to tackle severe equine asthma pathophysiology. However, to evaluate the potential of the DEmiRS to act as a non-invasive biomarker further research is needed. The 11 DEmiRs constitute future targets for future asthma research, helping to shed light on the still unknown molecular mechanisms underlying the disease. Since miRNAs are highly conserved and equine asthma shares striking similarities to human asthma, these new insights about the miRNA profile in the serum of asthmatic horses offers novel avenues for investigating the molecular pathology of human asthma [134,135].

## Figures and Tables

**Table 1:** Asthma-dependent DEmiRs. The list of significantly differentially expressed miRNAs affected by the asthmatic condition. The table includes mature equine miRNA ID, human mature miRNA homologue ID, DESeq2 normalized mean expression values, fold change in logarithmic scale and the false discovery rate (FDR) adjusted *p*-value reported by DESeq2 [51] and edgeR [52].

Mature Equine miRNA	Human Mature miRNA Homologue	Mean (DESeq2 Normalized) Expression	Log2 Fold Change	Adjusted *p* (DESeq2)	Adjusted *p* (edgeR)
eca-miR-128	hsa-miR-128-3p	526	−0.49	8.65 × 10^−4^	1.66 × 10^−3^
eca-miR-744	hsa-miR-744	168	−0.27	0.028	0.044
eca-miR-197	hsa-miR-197	633	−0.36	0.028	0.044
eca-miR-103	hsa-miR-103a-3p	51	0.34	0.028	0.044
eca-miR-107a	hsa-miR-107	51	0.34	0.028	0.044
eca-miR-30d	hsa-miR-30d	2073	−0.41	0.028	0.044
eca-miR-140-3p	hsa-miR-140-3p	251	0.37	0.033	0.044
eca-miR-7	hsa-miR-7-5p	70	0.47	0.035	0.06
eca-miR-361-3p	hsa-miR-361-3p	216	−0.39	0.035	0.044
eca-miR-148b-3p	hsa-miR-148b-3p	103	0.29	0.043	0.084
eca-miR-215	hsa-miR-215	823	0.44	0.05	0.11

**Table 2:** Top 10 enriched GeneGo pathway maps (based on MetaCore database). The pathway maps with the number of total genes involved and the number of target genes enriched for the pathway and the false discovery rate adjusted *p*-value (Adjusted *p*).

Maps	Total Genes in Pathway	Target Genes	Adjusted *p*
Development: Regulation of epithelial-to-mesenchymal transition (EMT)	64	11	1.024 × 10^−4^
Development: phosphatidylinositol (3,4,5)-triphosphate (PIP3) signaling in cardiac myocytes	47	9	2.703 × 10^−4^
Translation: Regulation of eukaryotic initiation factor 2 (EIF2) activity	39	8	4.293 × 10^−4^
Neurophysiological process: Dynein-dynactin motor complex in axonal transport in neurons	54	9	4.595 × 10^−4^
Regulation of glycogen synthase kinase 3 beta (GSK3β) in bipolar disorder	46	8	9.295 × 10^−4^
Development: hepatocyte growth factor (HGF)-dependent inhibition of transforming growth factor beta(TGFB)-induced EMT	34	7	9.295 × 10^−4^
Cell cycle: Regulation of G1/S transition (part 1)	38	7	1.730 × 10^−3^
Translation: Insulin regulation of translation	42	7	2.997 × 10^−3^
Development: WNT signaling pathway. Part 1. Degradation of beta-catenin in the absence WNT signaling	19	5	3.558 × 10^−3^
Development: Thrombopoietin-regulated cell processes	46	7	4.232 × 10^−3^

## Supplementary Materials

The following are available online at www.mdpi.com/2073-4425/8/12/383/s1. Supplementary file 1: Figure S1: Flowchart highlighting the changing sample size; Table S1: Table listing the horses of this study; Table S2: MiRDeep2 output table; Table S3: Identified putative novel equine miRNAs; Table S4: Identified putative novel equine miRNAs; Table S5: Table of miRNAs significantly affected by the level of haemolysis (analysis with DESeq2); Table S6: Table of miRNAs significantly affected by the level of haemolysis (analysis with edgeR); Table S7: Table outlining the statistics of the sample set used for differential miRNA expression analysis (*n* = 71); Figure S2: Bioanalyzer report. Supplementary file 2: Table S8: Experimentally validated target genes of DEmiRs.

## References

[1] Ansotegui I., Arruda L.K.P., Badellino H.A., Baena-Cagnani C.E., Bahna S.L., Baldacci S., Bel E., Bieber T.R.M., Bindslev-Jensen C., Blaiss M.S., Pawankar R., Holgate S.T., Canonica G.W., Lockey R.F., Blaiss M.S. (2013). White Book on Allergy: Update 2013.

[2] To T., Simatovic J., Zhu J., Feldman L., Dell S.D., Lougheed M.D., Licskai C., Gershon A. (2014). Asthma deaths in a large provincial health system: A 10-year population-based study. Ann. Am. Thorac. Soc..

[3] Gullach A.J., Risgaard B., Lynge T.H., Jabbari R., Glinge C., Haunsø S., Backer V., Winkel B.G., Tfelt-Hansen J. (2015). Sudden death in young persons with uncontrolled asthma—A nationwide cohort study in Denmark. BMC Pulm. Med..

[4] Torgerson D.G., Capurso D., Mathias R.A., Graves P.E., Hernandez R.D., Beaty T.H., Bleecker E.R., Raby B.A., Meyers D.A., Barnes K.C. (2012). Resequencing candidate genes implicates rare variants in asthma susceptibility. Am. J. Hum. Genet..

[5] Vercelli D. (2008). Discovering susceptibility genes for asthma and allergy. Nat. Rev. Immunol..

[6] Braun-Fahrlander C., Riedler J., Herz U., Eder W., Waser M., Grize L., Maisch S., Carr D., Gerlach F., Bufe A. (2002). Environmental exposure to endotoxin and its relation to asthma in school-age children. N. Engl. J. Med..

[7] Eisenbarth S.C., Piggott D.A., Huleatt J.W., Visintin I., Herrick C.A., Bottomly K. (2002). Lipopolysaccharide-enhanced, toll-like receptor 4-dependent T helper cell type 2 responses to inhaled antigen. J. Exp. Med..

[8] March M.E., Sleiman P.M., Hakonarson H. (2013). Genetic polymorphisms and associated susceptibility to asthma. Int. J. Gen. Med..

[9] Wenzel S.E. (2006). Asthma: Defining of the persistent adult phenotypes. Lancet.

[10] Ober C., Hoffjan S. (2006). Asthma genetics 2006: The long and winding road to gene discovery. Genes Immun..

[11] Moffatt M.F., Kabesch M., Liang L., Dixon A.L., Strachan D., Heath S., Depner M., von Berg A., Bufe A., Rietschel E. (2007). Genetic variants regulating ORMDL3 expression contribute to the risk of childhood asthma. Nature.

[12] Couëtil L.L., Cardwell J.M., Gerber V., Lavoie J.P., Léguillette R., Richard E.A. (2016). Inflammatory Airway Disease of Horses-Revised Consensus Statement. J. Vet. Intern. Med..

[13] Bullone M., Lavoie J.-P.P. (2015). Asthma “of horses and men”–How can equine heaves help us better understand human asthma immunopathology and its functional consequences?. Mol. Immunol..

[14] Herszberg B., Ramos-Barbón D., Tamaoka M., Martin J.G., Lavoie J.P. (2006). Heaves, an asthma-like equine disease, involves airway smooth muscle remodeling. J. Allergy Clin. Immunol..

[15] Pirie R.S., Dixon P.M., McGorum B.C. (2002). Evaluation of nebulised hay dust suspensions (HDS) for the diagnosis and investigation of heaves. 3: Effect of fractionation of HDS. Equine Vet. J..

[16] Lanz S., Gerber V., Marti E., Rettmer H., Klukowska-Rötzler J., Gottstein B., Matthews J.B., Pirie S., Hamza E. (2013). Effect of hay dust extract and cyathostomin antigen stimulation on cytokine expression by PBMC in horses with recurrent airway obstruction. Vet. Immunol. Immunopathol..

[17] Gerber V., Baleri D., Klukowska-Rötzler J., Swinburne J.E., Dolf G. (2009). Mixed inheritance of equine recurrent airway obstruction. J. Vet. Intern. Med..

[18] Swinburne J.E., Bogle H., Klukowska-Rötzler J., Drögemüller M., Leeb T., Temperton E., Dolf G., Gerber V. (2009). A whole-genome scan for recurrent airway obstruction in Warmblood sport horses indicates two positional candidate regions. Mamm. Genome.

[19] Marti E., Gerber H., Essich G., Oulehla J., Lazary S. (1991). The genetic basis of equine allergic diseases. 1. Chronic hypersensitivity bronchitis. Equine Vet J.

[20] Chen X., Ba Y., Ma L., Cai X., Yin Y., Wang K., Guo J., Zhang Y., Chen J., Guo X. (2008). Characterization of microRNAs in serum: A novel class of biomarkers for diagnosis of cancer and other diseases. Cell Res..

[21] Hashimoto Y., Akiyama Y., Yuasa Y. (2013). Multiple-to-Multiple Relationships between MicroRNAs and Target Genes in Gastric Cancer. PLoS ONE.

[22] Li J., Zhang Y., Li D., Liu Y., Chu D., Jiang X., Hou D., Zen K., Zhang C.Y. (2015). Small non-coding RNAs transfer through mammalian placenta and directly regulate fetal gene expression. Protein Cell.

[23] Liang H., Gong F., Zhang S., Zhang C.-Y.Y., Zen K., Chen X. (2014). The origin, function, and diagnostic potential of extracellular microRNAs in human body fluids. Wiley Interdiscip. Rev. RNA.

[24] Eissa N.T. (2015). The exosome in lung diseases: Message in a bottle. J. Allergy Clin. Immunol..

[25] Simpson L.J., Patel S., Bhakta N.R., Choy D.F., Brightbill H.D., Ren X., Wang Y., Pua H.H., Baumjohann D., Montoya M.M. (2014). A microRNA upregulated in asthma airway T cells promotes TH2 cytokine production. Nat. Immunol..

[26] Sethi A., Kulkarni N., Sonar S., Lal G. (2013). Role of miRNAs in CD4 T cell plasticity during inflammation and tolerance. Front. Genet..

[27] Okoye I.S., Czieso S., Ktistaki E., Roderick K., Coomes S.M., Pelly V.S., Kannan Y., Perez-Lloret J., Zhao J.L., Baltimore D. (2014). Transcriptomics identified a critical role for Th2 cell-intrinsic miR-155 in mediating allergy and antihelminth immunity. Proc. Natl. Acad. Sci. USA.

[28] Kim M.C., Lee S.W., Ryu D.Y., Cui F.J., Bhak J., Kim Y. (2014). Identification and Characterization of microRNAs in normal equine tissues by next generation sequencing. PLoS ONE.

[29] Pacholewska A., Mach N., Vaiman A., Schibler L., Barrey E., Gerber V. (2016). Novel equine tissue miRNAs and breed-related miRNA expressed in serum. BMC Genom..

[30] Buza T., Arick M., Wang H., Peterson D.G. (2014). Computational prediction of disease microRNAs in domestic animals. BMC Res. Notes.

[31] Van der Kolk J.H., Pacholewska A., Gerber V. (2015). The role of microRNAs in equine medicine: A review. Vet. Q..

[32] Kirschner M.B., Edelman J.J.B., Kao S.C.H., Vallely M.P., Van Zandwijk N., Reid G. (2013). The impact of hemolysis on cell-free microRNA biomarkers. Front. Genet..

[33] Pacholewska A., Jagannathan V., Drögemüller M., Klukowska-Rötzler J., Lanz S., Hamza E., Dermitzakis E.T., Marti E., Leeb T., Gerber V. (2015). Impaired Cell Cycle Regulation in a Natural Equine Model of Asthma. PLoS ONE.

[34] Ramseyer A., Gaillard C., Burger D., Straub R., Jost U., Boog C., Marti E., Gerber V. (2007). Effects of genetic and environmental factors on chronic lower airway disease in horses. J. Vet. Intern. Med..

[35] Laumen E., Doherr M.G., Gerber V. (2010). Relationship of horse owner assessed respiratory signs index to characteristics of recurrent airway obstruction in two Warmblood families. Equine Vet. J..

[36] Bosshard S., Gerber V. (2014). Evaluation of coughing and nasal discharge as early indicators for an increased risk to develop equine recurrent airway obstruction (RAO). J. Vet. Intern. Med..

[37] Pacholewska A., Drögemüller M., Klukowska-rötzler J., Lanz S., Hamza E., Dermitzakis E.T., Marti E., Gerber V., Leeb T., Jagannathan V. (2015). The transcriptome of equine peripheral blood mononuclear cells. PLoS ONE.

[38] Unger L., Fouché N., Leeb T., Gerber V., Pacholewska A. (2016). Optimized Method for Extracting Circulating Small RNAs from Long—Term Stored Equine Samples. Acta Vet. Scand..

[39] Kirschner M.B., Kao S.C., Edelman J.J., Armstrong N.J., Vallely M.P., van Zandwijk N., Reid G. (2011). Haemolysis during sample preparation alters microRNA content of plasma. PLoS ONE.

[40] Andrews S. (2010). FastQC—A Quality Control Tool for High Throughput Sequence Data. https://www.bioinformatics.babraham.ac.uk/projects/fastqc/.

[41] Martin M. (2011). Cutadapt removes adapter sequences from high-throughput sequencing reads. EMBnet J..

[42] European Nucleotide Archive. https://www.ebi.ac.uk/ena/data/view/PRJEB20494.

[43] Wade C.M., Giulotto E., Sigurdsson S., Zoli M., Gnerre S., Imsland F., Lear T.L., Adelson D.L., Bailey E., Bellone R.R. (2009). Genome Sequence, Comparative Analysis, and Population Genetics of the Domestic Horse. Science.

[44] Friedländer M.R., Mackowiak S.D., Li N., Chen W., Rajewsky N., Friedla M.R., Rajewsky N. (2012). MiRDeep2 accurately identifies known and hundreds of novel microRNA genes in seven animal clades. Nucleic Acids Res..

[45] Friedländer M.R., Chen W., Adamidi C., Maaskola J., Einspanier R., Knespel S., Rajewsky N. (2008). Discovering microRNAs from deep sequencing data using miRDeep. Nat. Biotechnol..

[46] Langmead B., Trapnell C., Pop M., Salzberg S. (2009). Ultrafast and memory-efficient alignment of short DNA sequences to the human genome. Genome Biol..

[47] Griffiths-Jones S., Grocock R.J., van Dongen S., Bateman A., Enright A.J. (2006). miRBase: microRNA sequences, targets and gene nomenclature. Nucleic Acids Res..

[48] Quinlan A.R., Hall I.M. (2010). BEDTools: A flexible suite of utilities for comparing genomic features. Bioinformatics.

[49] Koh W., Sheng C.T., Tan B., Lee Q.Y., Kuznetsov V., Kiang L.S., Tanavde V. (2010). Analysis of deep sequencing microRNA expression profile from human embryonic stem cells derived mesenchymal stem cells reveals possible role of let-7 microRNA family in downstream targeting of hepatic nuclear factor 4 alpha. BMC Genom..

[50] Kraft M.F. GitHub. https://github.com/MatthiasKraft/Serum_miRNA_GENES.

[51] Love M.I., Huber W., Anders S. (2014). Moderated estimation of fold change and dispersion for RNA-seq data with DESeq2. Genome Biol..

[52] Robinson M.D., McCarthy D.J., Smyth G.K. (2010). edgeR: A Bioconductor package for differential expression analysis of digital gene expression data. Bioinformatics.

[53] Anders S., McCarthy D.J., Chen Y., Okoniewski M., Smyth G.K., Huber W., Robinson M.D. (2013). Count-based differential expression analysis of RNA sequencing data using R and Bioconductor. Nat. Protoc..

[54] Lewis B.P., Burge C.B., Bartel D.P. (2005). Conserved seed pairing, often flanked by adenosines, indicates that thousands of human genes are microRNA targets. Cell.

[55] Kinsella R.J., Kähäri A., Haider S., Zamora J., Proctor G., Spudich G., Almeida-King J., Staines D., Derwent P., Kerhornou A. (2011). Ensembl BioMarts: A hub for data retrieval across taxonomic space. Database.

[56] Ashburner M., Ball C.A., Blake J.A., Botstein D., Butler H., Cherry J.M., Davis A.P., Dolinski K., Dwight S.S., Eppig J.T. (2000). Gene ontology: Tool for the unification of biology. The Gene Ontology Consortium. Nat. Genet..

[57] Nogales-Cadenas R., Carmona-Saez P., Vazquez M., Vicente C., Yang X., Tirado F., Carazo J.M., Pascual-Montano A. (2009). GeneCodis: Interpreting gene lists through enrichment analysis and integration of diverse biological information. Nucleic Acids Res..

[58] Tabas-Madrid D., Nogales-Cadenas R., Pascual-Montano A. (2012). GeneCodis3: A non-redundant and modular enrichment analysis tool for functional genomics. Nucleic Acids Res..

[59] Carmona-Saez P., Chagoyen M., Tirado F., Carazo J.M., Pascual-Montano A. (2007). GENECODIS: A web-based tool for finding significant concurrent annotations in gene lists. Genome Biol..

[60] Vlachos I.S., Paraskevopoulou M.D., Karagkouni D., Georgakilas G., Vergoulis T., Kanellos I., Anastasopoulos I.L., Maniou S., Karathanou K., Kalfakakou D. (2015). DIANA-TarBase v7.0: Indexing more than half a million experimentally supported miRNA:mRNA interactions. Nucleic Acids Res..

[61] Li H., Durbin R. (2009). Fast and accurate short read alignment with Burrows-Wheeler transform. Bioinformatics.

[62] Langmead B., Salzberg S.L. (2013). Fast gapped-read alignment with Bowtie 2. Nat. Methods.

[63] Nawrocki E.P., Burge S.W., Bateman A., Daub J., Eberhardt R.Y., Eddy S.R., Floden E.W., Gardner P.P., Jones T.A., Tate J. (2015). Rfam 12.0: Updates to the RNA families database. Nucleic Acids Res..

[64] Mueller R.S., Janda J., Jensen-Jarolim E., Rhyner C., Marti E. (2016). Allergens in veterinary medicine. Allergy Eur. J. Allergy Clin. Immunol..

[65] Lee S., Hwang S., Yu H.J., Oh D., Choi Y.J., Kim M.C., Kim Y., Ryu D.Y. (2016). Expression of microRNAs in horse plasma and their characteristic nucleotide composition. PLoS ONE.

[66] Pritchard C.C., Kroh E., Wood B., Arroyo J.D., Dougherty K.J., Miyaji M.M., Tait J.F., Tewari M. (2012). Blood cell origin of circulating microRNAs: A cautionary note for cancer biomarker studies. Cancer Prev. Res..

[67] Wang L.S., Li L., Li L., Chu S., Shiang K.D., Li M., Sun H.Y., Xu J., Xiao F.J., Sun G. (2015). MicroRNA-486 regulates normal erythropoiesis and enhances growth and modulates drug response in CML progenitors. Blood.

[68] Shaham L., Vendramini E., Ge Y., Goren Y., Birger Y., Tijssen M.R., McNulty M., Geron I., Schwartzman O., Goldberg L. (2015). MicroRNA-486-5p is an erythroid oncomiR of the myeloid leukemias of down syndrome. Blood.

[69] Rüegger S., Großhans H. (2012). MicroRNA turnover: When, how, and why. Trends Biochem. Sci..

[70] Hauser B., Zhao Y., Pang X., Ling Z., Myers E., Wang P., Califano J., Gu X. (2015). Functions of MiRNA-128 on the Regulation of Head and Neck Squamous Cell Carcinoma Growth and Apoptosis. PLoS ONE.

[71] Hu J., Cheng Y., Li Y., Jin Z., Pan Y., Liu G., Fu S., Zhang Y., Feng K., Feng Y. (2014). microRNA-128 plays a critical role in human non-small cell lung cancer tumourigenesis, angiogenesis and lymphangiogenesis by directly targeting vascular endothelial growth factor-C. Eur. J. Cancer.

[72] Sun J., Liao K., Wu X., Huang J., Zhang S., Lu X. (2015). Serum microRNA-128 as a biomarker for diagnosis of glioma. Int. J. Clin. Exp. Med..

[73] Adlakha Y.K., Saini N. (2013). miR-128 exerts pro-apoptotic effect in a p53 transcription-dependent and -independent manner via PUMA-Bak axis. Cell Death Dis..

[74] Adlakha Y.K., Khanna S., Singh R., Singh V.P., Agrawal A., Saini N. (2013). Pro-apoptotic miRNA-128-2 modulates ABCA1, ABCG1 and RXR[alpha] expression and cholesterol homeostasis. Cell Death Dis..

[75] Bartner L.R., Robinson N.E., Kiupel M., Tesfaigzi Y. (2006). Persistent mucus accumulation: A consequence of delayed bronchial mucous cell apoptosis in RAO-affected horses?. Am. J. Physiol. Lung Cell. Mol. Physiol..

[76] Moran G., Buechner-Maxwell V.A., Folch H., Henriquez C., Galecio J.S., Perez B., Carrasco C., Barria M. (2011). Increased apoptosis of CD4 and CD8 T lymphocytes in the airways of horses with recurrent airway obstruction. Vet. Res. Commun..

[77] Niedzwiedz A., Jaworski Z., Tykalowski B., Smialek M. (2014). Neutrophil and macrophage apoptosis in bronchoalveolar lavage fluid from healthy horses and horses with recurrent airway obstruction (RAO). BMC Vet. Res..

[78] Martinez-Nunez R.T., Bondanese V.P., Louafi F., Francisco-Garcia A.S., Rupani H., Bedke N., Holgate S., Howarth P.H., Davies D.E., Sanchez-Elsner T. (2014). A microRNA network dysregulated in asthma controls IL-6 production in bronchial epithelial cells. PLoS ONE.

[79] Rincon M., Irvin C.G. (2012). Role of IL-6 in asthma and other inflammatory pulmonary diseases. Int. J. Biol. Sci..

[80] Ordoñez C.L., Shaughnessy T.E., Matthay M.A., Fahy J.V. (2000). Increased neutrophil numbers and IL-8 levels in airway secretions in acute severe asthma: Clinical and biologic significance. Am. J. Respir. Crit. Care Med..

[81] Ainsworth D.M., Grönig G., Matychak M.B., Young J., Wagner B., Erb H.N., Antczak D.F. (2003). Recurrent airway obstruction (RAO) in horses is characterized by IFNG and IL-8 production in bronchoalveolar lavage cells. Vet. Immunol. Immunopathol..

[82] Berndt A., Derksen F.J., Venta P.J., Karmaus W., Ewart S., Robinson N.E. (2009). Expression of toll-like receptor 2 mRNA in bronchial epithelial cells is not induced in RAO-affected horses. Equine Vet. J..

[83] Ainsworth D.M., Wagner B., Franchini M., Grünig G., Erb H.N., Tan J.Y. (2006). Time-dependent alterations in gene expression of interleukin-8 in the bronchial epithelium of horses with recurrent airway obstruction. Am. J. Vet. Res..

[84] Chu D.K., Al-Garawi A., Llop-Guevara A., Pillai R.A., Radford K., Shen P., Walker T.D., Goncharova S., Calhoun W.J., Nair P. (2015). Therapeutic potential of anti-IL-6 therapies for granulocytic airway inflammation in asthma. Allergy Asthma Clin. Immunol..

[85] Smolen J.S., Maini R.N. (2006). Interleukin-6: A new therapeutic target. Arthritis Res. Ther..

[86] Martin J., Jenkins R.H., Bennagi R., Krupa A., Phillips A.O., Bowen T., Fraser D.J. (2011). Post-transcriptional regulation of transforming growth factor beta-1 by microRNA-744. PLoS ONE.

[87] Debrue M., Hamilton E., Joubert P., Lajoie-Kadoch S., Lavoie J.P. (2005). Chronic exacerbation of equine heaves is associated with an increased expression of interleukin-17 mRNA in bronchoalveolar lavage cells. Vet. Immunol. Immunopathol..

[88] Newcomb D.C., Peebles R.S. (2013). Th17-mediated inflammation in asthma. Curr. Opin. Immunol..

[89] Wang H., Su X., Yang M., Chen T., Hou J., Li N., Cao X. (2015). Reciprocal control of miR-197 and IL-6/STAT3 pathway reveals miR-197 as potential therapeutic target for hepatocellular carcinoma. Oncoimmunology.

[90] Yang X.O., Panopoulos A.D., Nurieva R., Seon H.C., Wang D., Watowich S.S., Dong C. (2007). STAT3 regulates cytokine-mediated generation of inflammatory helper T cells. J. Biol. Chem..

[91] Chalmin F., Mignot G., Bruchard M., Chevriaux A., Végran F., Hichami A., Ladoire S., Derangère V., Vincent J., Masson D. (2012). Stat3 and Gfi-1 Transcription Factors Control Th17 Cell Immunosuppressive Activity via the Regulation of Ectonucleotidase Expression. Immunity.

[92] Takagi R., Higashi T., Hashimoto K., Nakano K., Mizuno Y., Okazaki Y., Matsushita S. (2008). B cell chemoattractant CXCL13 is preferentially expressed by human Th17 cell clones. J. Immunol..

[93] Eddens T., Elsegeiny W., Garcia-Hernadez M.L., Castillo P., Trevejo-Nunez G., Serody K., Campfield B.T., Khader S.A., Chen K., Rangel-Moreno J. (2017). Pneumocystis-Driven Inducible Bronchus-Associated Lymphoid Tissue Formation Requires Th2 and Th17 Immunity. Cell Rep..

[94] Elliot J.G., Jensen C.M., Mutavdzic S., Lamb J.P., Carroll N.G., James A.L. (2004). Aggregations of lymphoid cells in the airways of nonsmokers, smokers, and subjects with asthma. Am. J. Respir. Crit. Care Med..

[95] Damsker J.M., Hansen A.M., Caspi R.R. (2010). Th1 and Th17 cells: Adversaries and collaborators. Ann. N. Y. Acad. Sci..

[96] Irvin C., Zafar I., Good J., Rollins D., Christianson C., Gorska M.M., Martin R.J., Alam R. (2014). Increased frequency of dual-positive TH2/TH17 cells in bronchoalveolar lavage fluid characterizes a population of patients with severe asthma. J. Allergy Clin. Immunol..

[97] Takahashi Y., Forrest A.R.R., Maeno E., Hashimoto T., Daub C.O., Yasuda J. (2009). MiR-107 and MiR-185 can induce cell cycle arrest in human non small cell lung cancer cell lines. PLoS ONE.

[98] Xia W., Ni J., Zhuang J., Qian L., Wang P., Wang J. (2016). MiR-103 regulates hepatocellular carcinoma growth by targeting AKAP12. Int. J. Biochem. Cell Biol..

[99] Badalà F., Nouri-mahdavi K., Raoof D.A. (2008). Expansion of an atypical NK cell subset in mouse models of SLE. Computer (Long. Beach. Calif)..

[100] Ballabio E., Mitchell T., van Kester M.S., Taylor S., Dunlop H.M., Tosi I., Vermeer M.H., Tramonti D., Saunders N.J., Boultwood J. (2013). MicroRNA expression in Sézary syndrome: Identification, function, and diagnostic potential. Blood.

[101] Akbas F., Coskunpinar E., Yildiz P. (2012). Analysis of serum micro-rnas as potential biomarker in chronic obstructive. Exp. Lung Res..

[102] Hong C.F., Lin S.Y., Chou Y.T., Wu C.W. (2016). MicroRNA-7 compromises p53 protein-dependent apoptosis by controlling the expression of the chromatin remodeling factor SMARCD1. J. Biol. Chem..

[103] Porto I.O.P., Mendes-Junior C.T., Felício L.P., Georg R.C., Moreau P., Donadi E.A., Chies J.A.B., Castelli E.C. (2015). MicroRNAs targeting the immunomodulatory HLA-G gene: A new survey searching for microRNAs with potential to regulate HLA-G. Mol. Immunol..

[104] Tan Z., Randall G., Fan J., Camoretti-Mercado B., Brockman-Schneider R., Pan L., Solway J., Gern J.E., Lemanske R.F., Nicolae D. (2008). Allele-Specific Targeting of microRNAs to HLA-G and Risk of Asthma (PII:S0002-9297(07)63059-6). Am. J. Hum. Genet..

[105] Enomoto Y., Takagi R., Naito Y., Kiniwa T., Tanaka Y., Hamada-Tsutsumi S., Kawano M., Matsushita S., Ochiya T., Miyajima A. (2017). Identification of the novel 3′ UTR sequences of human IL-21 mRNA as potential targets of miRNAs. Sci. Rep..

[106] Sun Y., Pan J., Mao S., Jin J. (2014). IL-17/miR-192/IL-17Rs regulatory feedback loop facilitates multiple myeloma progression. PLoS ONE.

[107] McGeachie M.J., Davis J.S., Kho A.T., Dahlin A., Sordillo J.E., Sun M., Lu Q., Weiss S.T., Tantisira K.G. (2017). Asthma remission: Predicting future airways responsiveness using an miRNA network. J. Allergy Clin. Immunol..

[108] Pereira-Leal J.B., Seabra M.C. (2001). Evolution of the Rab family of small GTP-binding proteins. J. Mol. Biol..

[109] Seto S., Tsujimura K., Koide Y. (2011). Rab GTPases Regulating Phagosome Maturation Are Differentially Recruited to Mycobacterial Phagosomes. Traffic.

[110] Ding L., Abebe T., Beyene J., Wilke R.A., Goldberg A., Woo J.G., Martin L.J., Rothenberg M.E., Rao M., Hershey G.K.K. (2013). Rank-based genome-wide analysis reveals the association of Ryanodine receptor-2 gene variants with childhood asthma among human populations. Hum. Genom..

[111] Probert K., Miller S., Kheirallah A.K., Hall I.P. (2015). Developmental genetics of the COPD lung. COPD Res. Pract..

[112] Tang W., Kowgier M., Loth D.W., Soler Artigas M., Joubert B.R., Hodge E., Gharib S.A., Smith A.V., Ruczinski I., Gudnason V. (2014). Large-Scale Genome-Wide Association Studies and Meta-Analyses of Longitudinal Change in Adult Lung Function. PLoS ONE.

[113] Szabo S.J., Kim S.T., Costa G.L., Zhang X., Fathman C.G., Glimcher L.H. (2000). A novel transcription factor, T-bet, directs Th1 lineage commitment. Cell.

[114] Mullen A.C., Hutchins A.S., High F.A., Lee H.W., Sykes K.J., Chodosh L.A., Reiner S.L. (2002). Hlx is induced by and genetically interacts with T-bet to promote heritable T(H)1 gene induction. Nat. Immunol..

[115] Casaca V.I., Illi S., Suttner K., Schleich I., Ballenberger N., Klucker E., Turan E., von Mutius E., Kabesch M., Schaub B. (2012). TBX21 and HLX1 polymorphisms influence cytokine secretion at birth. PLoS ONE.

[116] Murphy T.M., Wong C.C.Y., Arseneault L., Burrage J., Macdonald R., Hannon E., Fisher H.L., Ambler A., Moffitt T.E., Caspi A. (2015). Methylomic markers of persistent childhood asthma: A longitudinal study of asthma-discordant monozygotic twins. Clin. Epigenet..

[117] Suttner K., Rosenstiel P., Depner M., Schedel M., Pinto L.A., Ruether A., Adamski J., Klopp N., Illig T., Vogelberg C. (2009). TBX21 gene variants increase childhood asthma risk in combination with HLX1 variants. J. Allergy Clin. Immunol..

[118] Suttner K., Ruoss I., Rosenstiel P., Depner M., Pinto L.A., Schedel M., Adamski J., Illig T., Schreiber S., von Mutius E., Kabesch M. (2009). HLX1 gene variants influence the development of childhood asthma. J. Allergy Clin. Immunol..

[119] Wenzel S.E. (2012). Asthma phenotypes: The evolution from clinical to molecular approaches. Nat. Med..

[120] Raundhal M., Morse C., Khare A., Oriss T.B., Milosevic J., Trudeau J., Huff R., Pilewski J., Holguin F., Kolls J. (2015). High IFN-γ and low SLPI mark severe asthma in mice and humans. J. Clin. Investig..

[121] Hackett T.-L. (2012). Epithelial–mesenchymal transition in the pathophysiology of airway remodelling in asthma. Curr. Opin. Allergy Clin. Immunol..

[122] Ijaz T., Pazdrak K., Kalita M., Konig R., Choudhary S., Tian B., Boldogh I., Brasier A.R. (2014). Systems biology approaches to understanding Epithelial Mesenchymal Transition (EMT) in mucosal remodeling and signaling in asthma. World Allergy Organ. J..

[123] Han J.M., Patterson S.J., Levings M.K. (2012). The Role of the PI3K Signaling Pathway in CD4+ T Cell Differentiation and Function. Front. Immunol..

[124] Wang J., Li F., Yang M., Wu J., Zhao J., Gong W., Liu W., Bi W., Dong L. (2014). FIZZ1 promotes airway remodeling through the PI3K/Akt signaling pathway in asthma. Exp. Ther. Med..

[125] Sheller J.R., Polosukhin V.V., Mitchell D., Cheng D.-S., Peebles R.S., Blackwell T.S. (2009). NF-kB Induction in Airway Epithelium Increases Lung Inflammation in Allergen Challenged Mice. Exp. Lung Res..

[126] Graham J.A., Fray M., de Haseth S., Lee K.M., Lian M.M., Chase C.M., Madsen J.C., Markmann J., Benichou G., Colvin R.B. (2010). Suppressive Regulatory T Cell Activity Is Potentiated by Glycogen Synthase Kinase 3 beta Inhibition. J. Biol. Chem..

[127] Fu R., Li J., Zhong H., Yu D., Zeng X., Deng M., Sun Y., Wen W., Li H. (2014). Broncho-vaxom attenuates allergic airway inflammation by restoring GSK3β-related T regulatory cell insufficiency. PLoS ONE.

[128] Coutaz M., Hurrell B.P., Auderset F., Wang H., Siegert S., Eberl G., Ho P.-C., Radtke F., Tacchini-Cottier F. (2016). Notch regulates Th17 differentiation and controls trafficking of IL-17 and metabolic regulators within Th17 cells in a context-dependent manner. Sci. Rep..

[129] Hernandez J.B., Chang C., LeBlanc M., Grimm D., Le Lay J., Kaestner K.H., Zheng Y., Montminy M. (2015). The CREB/CRTC2 pathway modulates autoimmune disease by promoting Th17 differentiation. Nat. Commun..

[130] Lane S.J., Adcock I.M., Richards D., Hawrylowicz C., Barnes P.J., Tak H.L. (1998). Corticosteroid-resistant bronchial asthma is associated with increased c-fos expression in monocytes and T lymphocytes. J. Clin. Investig..

[131] Janssen-Heininger Y.M.W., Poynter M.E., Aesif S.W., Pantano C., Ather J.L., Reynaert N.L., Ckless K., Anathy V., van der Velden J., Irvin C.G. (2009). Nuclear Factor _k_B, airway epithelium, and asthma: Avenues for redox control. Proc. Am. Thorac. Soc..

[132] Ren Y., Su X., Kong L., Li M., Zhao X., Yu N., Kang J. (2016). Therapeutic effects of histone deacetylase inhibitors in a murine asthma model. Inflamm. Res..

[133] DuPage M., Bluestone J.A. (2016). Harnessing the plasticity of CD4+ T cells to treat immune-mediated disease. Nat. Rev. Immunol..

[134] Leclere M., Lavoie-Lamoureux A., Lavoie J.P. (2011). Heaves, an asthma-like disease of horses. Respirology.

[135] Lee C.-T., Risom T., Strauss W.M. (2007). Evolutionary conservation of microRNA regulatory circuits: An examination of microRNA gene complexity and conserved microRNA-target interactions through metazoan phylogeny. DNA Cell Biol..

